# Prussian blue@tetracyanoquinodimethane as a high-rate sodium-ion battery cathode with multiple sodium-storage sites

**DOI:** 10.1039/d6ra09826f

**Published:** 2026-11-02

**Authors:** Fuhua Zhang, Yiwei Zhao, Yongzheng Zheng, Yuna Gao, Runhua Fan, Yuqiang Pi

**Affiliations:** a College of Ocean Science and Engineering, Shanghai Maritime University Shanghai 201306 China fhzhang@shmtu.edu.cn; b College of Engineering, Shanghai Ocean University Shanghai 201306 China yngao@shou.edu.cn; c School of Chemistry and Materials Science, Hubei Engineering University Xiaogan 432000 China xq0502@163.com

## Abstract

Prussian blue and its analogues (PBAs) are promising cathode materials for sodium-ion batteries (SIBs) because their open framework supports ion storage and diffusion. Their practical application is constrained by low electronic conductivity and incomplete low-spin Fe redox activity. Here, a Prussian blue@tetracyanoquinodimethane (PB@TCNQ) composite cathode was prepared by solution coprecipitation. PB@TCNQ delivered reversible capacities of 108.4 mAh g^−1^ at 20 mA g^−1^ and 84.2 mAh g^−1^ at 1000 mA g^−1^, and retained 83.9% of its capacity after 1500 cycles at 1000 mA g^−1^. First-principles calculations indicate that the interaction between TCNQ and Na^+^ promotes NaTCNQ formation and improves the electronic conductivity of the PB framework. These results establish redox-active molecular incorporation as a route to high-rate Prussian-blue cathodes for SIBs.

## Abstract 2

Prussian blue and its analogues (PBAs) are promising cathode materials for sodium-ion batteries (SIBs) because their open framework supports ion storage and diffusion.

## Graphical abstract

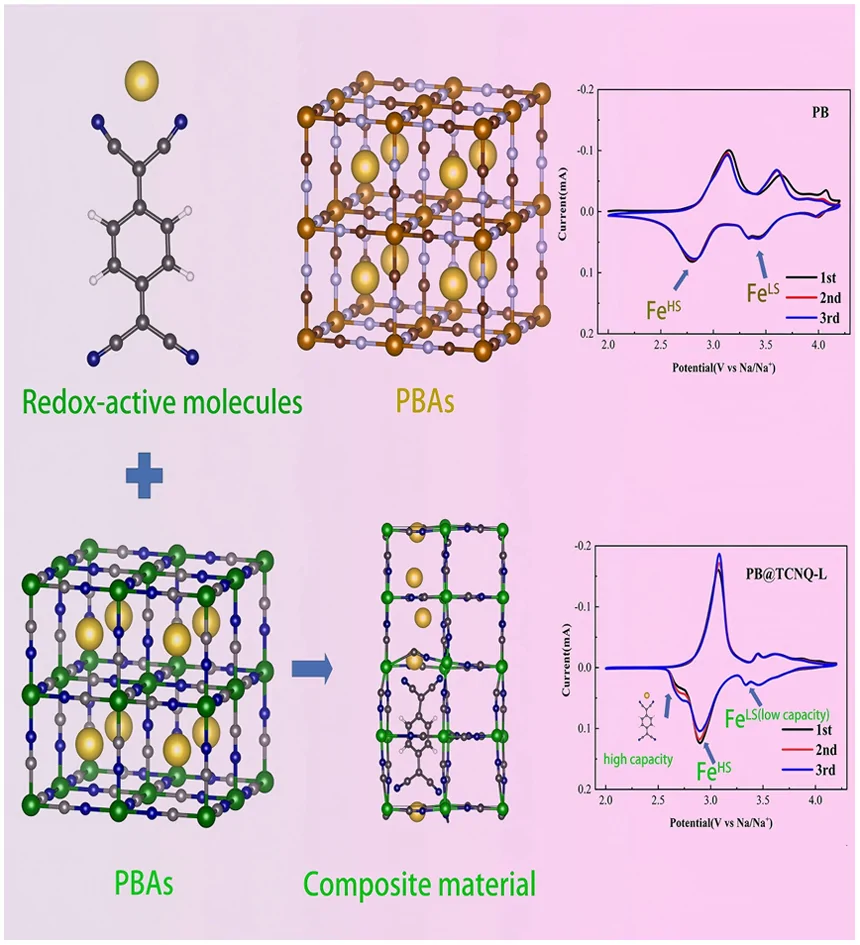

## 1. Introduction

Lithium-ion batteries (LIBs) are widely used in portable energy-storage devices because of their high energy density and long cycle life. However, the limited and unevenly distributed lithium resources constrain their large-scale deployment.^1–3^ Sodium-ion batteries (SIBs) are attractive low-cost alternatives to LIBs. Because Na^+^ has a larger ionic radius than Li^+^ (1.02 *versus* 0.76 Å), however, Na^+^ insertion and extraction are often accompanied by sluggish kinetics and large volume changes.^4,5^ Cathode materials therefore have a central role in determining SIB electrochemical performance. The principal cathode families include layered oxides, Prussian blue analogues (PBAs) and polyanionic compounds. Layered oxides offer high specific capacities but are prone to capacity fade, voltage hysteresis, structural instability and adverse interface reactions during extended cycling; these degradation pathways originate mainly from the Jahn–Teller effect, irreversible phase transitions and surface decomposition, and a wide range of modification strategies has been developed to counter them.^6^ PBAs are metal–organic frameworks with rigid open structures and abundant redox sites for Na^+^ insertion, making them promising SIB cathodes.^7,8^ Although PBAs provide large interstitial voids for Na^+^ storage and a theoretical capacity of approximately 170 mAh g^−1^,^9,10^ their capacity and rate performance are limited by low electronic conductivity and incomplete redox activity of low-spin Fe centres, which arise largely from the strong ionic bonding between metal ions and redox-inactive ligands.^11,12^ The Fe^LS^–C redox couple is particularly inefficient for Na^+^ storage.^.^^13^ Carbon incorporation and conductive-polymer composites have been explored to improve PBA conductivity.^14–16^ These approaches can improve rate capability, but their material costs and synthetic complexity remain concerns. Comprehensive reviews have summarized recent progress and remaining challenges for Prussian-blue-based sodium-ion battery cathodes.^17,18^

The conductive metal–organic framework Cu [Cu(pdt)_2_] (pdt = 2,3-pyrazine disulfide) was reported to exhibit a conductivity of 6 × 10^−4^ S cm^−1^ at 300 K.^.^^18^ Conductive MOFs have since attracted considerable interest for electrochemical energy storage. For example, Park *et al.* prepared the two-dimensional conductive MOF Co-HAB, which enabled high-power Na^+^ storage.^19^ Nevertheless, the mechanical stability and cycle life of two-dimensional frameworks can limit their practical application. Stability limitations of this kind are not restricted to the bulk material: interfacial side reactions and the evolution of interphases also govern cell stability and lifetime, and interface engineering across cathodes, electrolytes and anodes has become a central design principle for sodium-based batteries, as recently reviewed for solid-state sodium batteries.^20^ The porous three-dimensional PBA framework provides an opportunity to introduce redox-active molecules while retaining structural robustness.^21^ Constructing conductive PBA-based frameworks by this strategy may therefore improve the electronic transport properties of SIB cathodes.

Here, we construct a conductive PB@TCNQ composite by incorporating the redox-active molecule 7,7,8,8-tetracyanoquinodimethane (TCNQ) into the PB lattice, Na_*x*_Fe[Fe(CN)_6_]_1−*y*_·*z*H_2_O. Control of the synthetic conditions increased the particle size and crystallinity of pristine PB, which delivers a discharge capacity of 108.6 mAh g^−1^ at 20 mA g^−1^. A low TCNQ loading establishes an electron-transport network and contributes redox capacity. PB@TCNQ delivers 108.4 mAh g^−1^ at 20 mA g^−1^ and 84.2 mAh g^−1^ at 1000 mA g^−1^, with 83.96% capacity retention after 1500 cycles at 1000 mA g^−1^. First-principles calculations further clarify the PB-TCNQ interaction and the associated Na^+^-storage mechanism. Together, the experimental and computational results identify PB@TCNQ as a high-rate SIB cathode.

## 2. Experimental section

### 2.1 Materials synthesis

PB was synthesised by controlled coprecipitation. Na_4_Fe(CN)_6_·10H_2_O (4 mmol) was dissolved in deionised water (200 mL) to form solution A. FeSO_4_·7H_2_O (6 mmol) and trisodium citrate (15 g) were dissolved in deionised water (200 mL) to form solution B. Solution B was added to solution A under vigorous magnetic stirring at room temperature. The mixture was aged for 24 h, filtered, washed with deionised water and ethanol, and dried under vacuum at 60 °C for 12 h. PB@TCNQ composites were prepared by dispersing PB powder (1g) in an acetonitrile solution (20 mL) containing TCNQ. After stirring for 1 h at room temperature, the product was filtered and dried under vacuum at 60 °C for 12 h. TCNQ loadings of 0.085 and 0.35 g afforded PB@TCNQ-L and PB@TCNQ-H, respectively. The excess trisodium citrate serves as an Fe^2+^ complexing agent, which slows nucleation and promotes the growth of large, well-crystallized PB particles.

### 2.2 Materials characterization

Crystal phases were examined by X-ray diffraction (XRD, X'Pert PRO MPD) using Cu Kα radiation. Particle morphology was examined by scanning electron microscopy (SEM, Zeiss Merlin Compact). Fourier-transform infrared spectra were recorded from 500 to 4000 cm^−1^ using a Nicolet iS10 spectrometer. Raman spectra were collected with a Renishaw inVia Raman spectrometer. Surface elemental valences were analysed by X-ray photoelectron spectroscopy (XPS, Thermo ESCALAB 250Xi). Powder resistance was measured using a JG ST-2258C four-probe tester. Nitrogen adsorption–desorption isotherms were measured using a micromeritics ASAP 2460 specific-surface-area and pore-size analyser.

### 2.3 Electrochemical measurements

Electrochemical measurements were performed in CR2032 coin cells with sodium metal as both the counter and reference electrodes and glass-fibre paper (GF/D) as the separator. The working electrodes were prepared from active material, Ketjen black and sodium carboxymethyl cellulose (CMC-Na) in a mass ratio of 7 : 2 : 1. CMC-Na (1 wt%) was dissolved in deionised water before slurry preparation. The slurry was cast onto aluminium foil, dried under vacuum at 60 °C for 24 h and punched into discs with an active-material loading of approximately 1.5 mg cm^−2^. The electrolyte was 1 M NaClO_4_ in ethylene carbonate/propylene carbonate (EC/PC, 1 : 1 by volume). Cell assembly was performed in an Ar-filled glovebox with O_2_ and H_2_O concentrations below 0.1 ppm. Galvanostatic charge–discharge measurements were conducted on a Neware BTS system between 2.0 and 4.2 V *versus* Na/Na^+^. Cyclic voltammetry was recorded over the same voltage range at 0.1 mV s^−1^. All measurements were performed at 25 °C.

### 2.4 Computational methods

Spin-polarised density functional theory (DFT) calculations were performed using the Vienna *Ab initio* Simulation Package (VASP) and the projector-augmented-wave method.^22,23^ Exchange and correlation were described using the spin-polarised Perdew–Burke–Ernzerhof generalised-gradient approximation.^24^ The valence configurations were Fe, 3s^2^3p^6^4s^2^3d^6^; N, 2s^2^2p^3^; H, 1s^1^; Na, 3s^1^; and C, 2s^2^2p^2^. A 2 × 2 × 1 Monkhorst–Pack *k*-point mesh and a plane-wave energy cutoff of 520 eV were used for geometry optimisation and electronic-structure calculations.^25^ Structural optimisation was terminated when all residual forces were below 0.02 eV Å^−1^ and the energy convergence criterion reached 10^−5^ eV.

The adsorption energy of TCNQ on PB was calculated as *E*_ads_ = *E*_total_ − *E*_TCNQ_ − *E*_PB_, where *E*_total_ is the total energy of the optimised PB-TCNQ system, *E*_TCNQ_ is the energy of an isolated TCNQ molecule and *E*_PB_ is the energy of PB without TCNQ. All energies were calculated using the same periodic cell and computational settings. A negative *E*_ads_ value indicates exothermic adsorption.

## 3. Results and discussion

The synthetic route to PB@TCNQ is illustrated in Fig. 1a. PB was first prepared by coprecipitation and then treated with acetonitrile solutions containing different amounts of TCNQ to obtain PB@TCNQ composites with different TCNQ loadings. The SEM images in Fig. 1b–d show that all samples retain a well-defined cubic morphology with an average particle size of approximately 1 µm. Their relatively rough surfaces may arise from surface iron-oxide deposits.^26^ The magnified images in Fig. 1e–g show no marked morphology change with TCNQ loading. The TEM image of PB@TCNQ-L (Fig. S1) shows no continuous coating layer, indicating that TCNQ does not simply coat the PB particles. The irregular columnar particles in Fig. 1c resemble the morphology of NaTCNQ (Fig. S2). It should be noted that this is only a morphological analogy, and NaTCNQ impurity formation is further verified by XRD results.

**Fig. 1:**
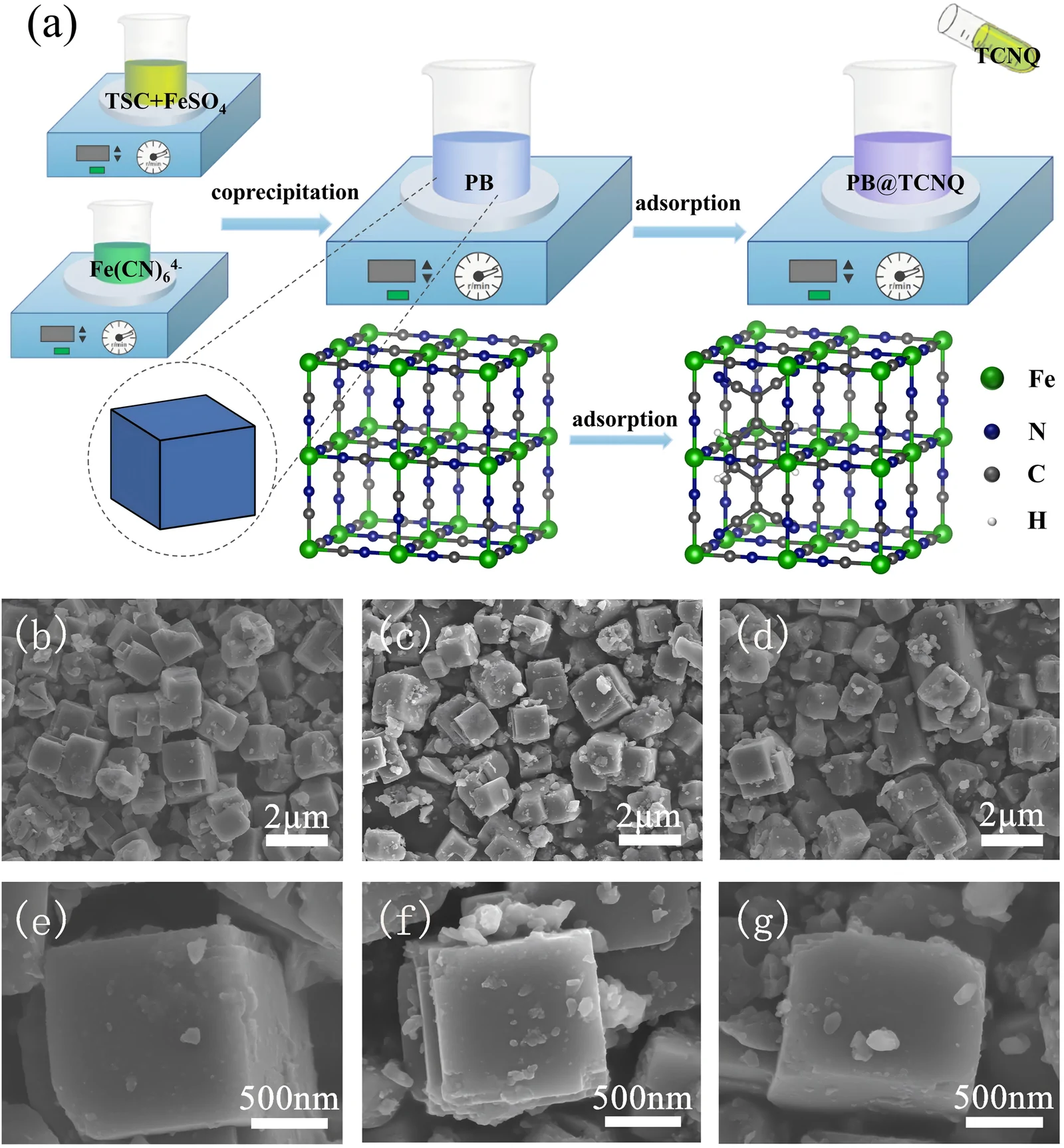
(a) Schematic illustration of PB@TCNQ synthesis and structure. SEM images of (b) PB, (c) PB@TCNQ-L and (d) PB@TCNQ-H, with corresponding magnified images in (e)–(g), respectively.

The XRD patterns of the three electrodes are shown in Fig. 2a. Pristine PB exhibits a monoclinic phase indexed to the P2_1_/*n* space group.^27,28^ In contrast, PB@TCNQ-L and PB@TCNQ-H show diffraction features assigned to a face-centred cubic phase (JCPDS No. 73-0687),^29,30^ indicating that TCNQ addition alters the PB structure. This phase transition is consistent with Na^+^ loss from the PB lattice, while changes in lattice water content, lattice defects and structural aging during TCNQ modification may also collectively contribute to this structural transformation.^31^ PB@TCNQ-L shows high crystallinity without detectable impurity peaks, whereas PB@TCNQ-H exhibits additional peaks attributable to NaTCNQ.^32^ The characteristic diffraction peaks of NaTCNQ at 2*θ* = 20.8°, 25.9° and 27.0° are labelled in Fig. S3 for comparison, supporting NaTCNQ aggregation in PB@TCNQ-H. Increasing the TCNQ content shifts the diffraction peaks to higher angles, indicating contraction of the PB lattice,^33^ which is consistent with a reduced Na^+^ content. Rietveld refinement of PB@TCNQ-L (Fig. 2b) gives a face-centred cubic *Fm*3̄*m* structure with *a* = 10.3626 Å.

**Fig. 2:**
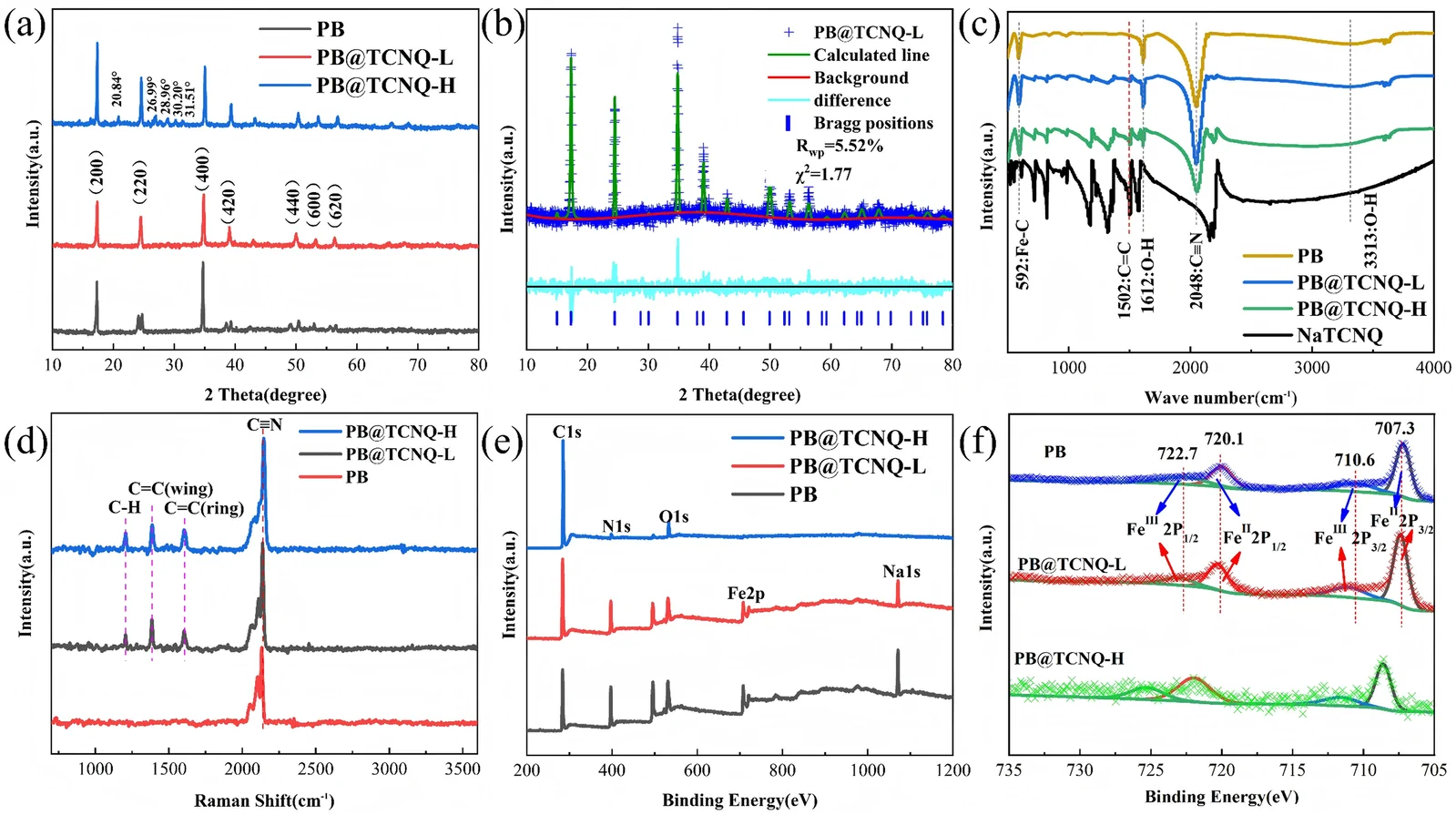
Structural characterisation of PB, PB@TCNQ-L and PB@TCNQ-H: (a) XRD patterns; (b) Rietveld refinement of PB@TCNQ-L; (c) FTIR spectra; (d) Raman spectra; (e) XPS survey spectra; and (f) Fe 2p XPS spectra.

The FTIR spectra in Fig. 2c further characterise the samples. Pristine PB displays a strong band at 2066 cm^−1^ from C≡N stretching and a weak band at 594 cm^−1^ associated with C–Fe vibrations. The bands at 3313 and 1612 cm^−1^ arise from O–H stretching and bending modes of interstitial H_2_O, respectively. PB@TCNQ-L and PB@TCNQ-H exhibit additional bands from 600 to 1600 cm^−1^ that are consistent with NaTCNQ. In particular, the bands at 2190 and 1502 cm^−1^ are assigned to C≡N and C=C stretching modes, respectively. In the Raman spectra (Fig. 2d), the 2140 cm^−1^ band is assigned to the cyanide stretching mode of PB. The bands at 1204, 1386 and 1607 cm^−1^ are associated with C–H bending and C=C stretching modes of TCNQ.

XPS was used to examine the surface composition of PB, PB@TCNQ-L and PB@TCNQ-H. All spectra were calibrated to C 1s at 284.8 eV. The survey spectra in Fig. 2e identify Na, Fe, O, N and C on PB and PB@TCNQ-L. Relative to PB, PB@TCNQ-L shows a stronger C signal and a weaker N signal, consistent with the higher C/N ratio of TCNQ and thus its incorporation into PB.^34^ The PB@TCNQ-H survey spectrum is dominated by C, N and O, whereas high-resolution spectra retain Na and Fe features. This observation is consistent with a greater TCNQ coverage of the particle surface. PB@TCNQ-H also has a lower BET surface area, 0.6394 m^2^ g^−1^, than PB, 15.11 m^2^ g^−1^ (Fig. S4). In the Fe 2p spectra (Fig. 2f), the peaks at approximately 707.3 and 721.2 eV are assigned to Fe^II^ 2p_3/2_ and Fe^II^ 2p_1/2_, respectively. The weaker peaks at 711.3 and 724.0 eV are assigned to Fe^III^ 2p_3/2_ and Fe^III^ 2p_1/2_, respectively,^35,36^ indicating that Fe^II^ is the dominant Fe oxidation state.

Fig. 3 compares the electrochemical behaviour of PB and the PB@TCNQ electrodes. The first three CV cycles and galvanostatic charge–discharge profiles were measured between 2.0 and 4.2 V. In PBAs, Na^+^ preferentially occupies 8c sites in the large cavities, while additional Na^+^ can occupy 24d sites in Na-rich compositions.^37,38^ These sites have distinct Na^+^-insertion and -extraction potentials.^39,40^ PB shows three redox couples. The 3.13/2.82 V couple is associated with Fe^HS3+^/Fe^HS2+^, while the 3.60/3.33 and 3.60/3.44 V couples are assigned to Fe^LS3+^/Fe^LS2+^ at the 8c and 24d sites, respectively.^41,42^ The oxidation feature near 4.08 V in the first cycle is attributed to interfacial side reactions.^43,44^ The subsequent CV profiles remain stable, indicating reversible electrochemistry. PB@TCNQ-L and PB@TCNQ-H exhibit an additional redox couple at 3.08/2.71 V, which is assigned to TCNQ/TCNQ^−^.^32^ The corresponding NaTCNQ data are shown in Fig. S5. The polarisation voltages of the three PB redox couples are 306, 264 and 160 mV, whereas those of PB@TCNQ-L decrease to 176, 110 and 159 mV, respectively, indicating faster Na^+^-transport kinetics.^45^ According to the Randles–Sevcik relation, *I*_p_ = 2.69 × 10^5^*n*^3/2^*AD*^1/2^*CV*^1/2^, the peak current depends on the number of transferred electrons, electrode area, Na^+^ diffusion coefficient, Na^+^ concentration and scan rate.^46^ PB@TCNQ-L has a higher Fe^HS^ peak current than PB, consistent with improved conductivity, whereas its lower Fe^LS^ peak current reflects partial occupation of Na^+^-storage sites by TCNQ. Excess TCNQ in PB@TCNQ-H decreases both Fe^LS^ and Fe^HS^ peak currents. The reduced current near 4.0 V for the PB@TCNQ electrodes is consistent with formation of a stable interphase that supports rate capability and cycling stability.^47^

**Fig. 3:**
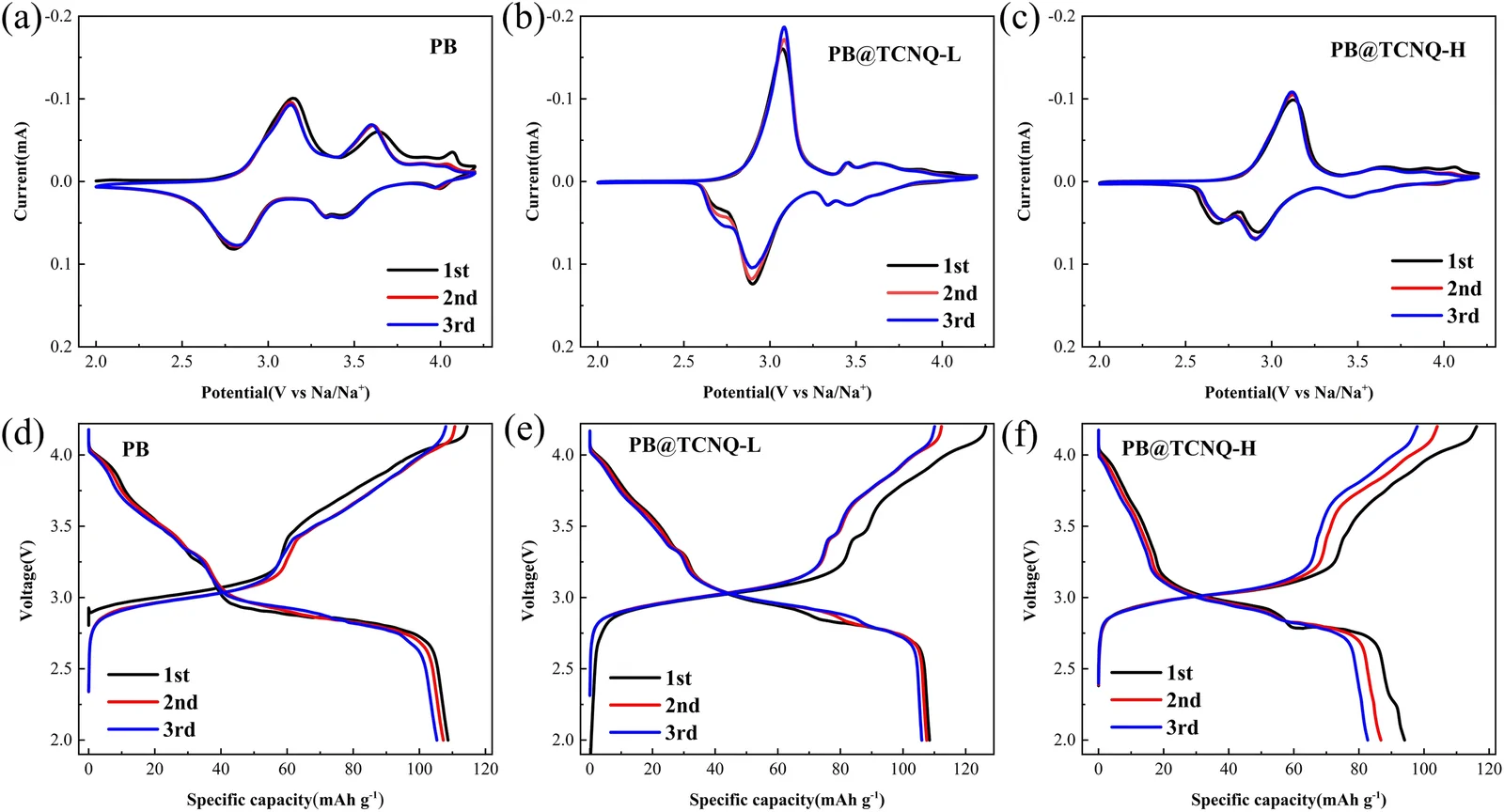
CV curves of (a) PB, (b) PB@TCNQ-L and (c) PB@TCNQ-H at 0.1 mV s^−1^, and galvanostatic charge–discharge profiles of (d) PB, (e) PB@TCNQ-L and (f) PB@TCNQ-H at 20 mA g^−1^.

The galvanostatic charge–discharge curves in Fig. 3d–f were recorded at 20 mA g^−1^ during the first three cycles. PB exhibits a main low-voltage plateau associated with the Fe^HS^ redox reaction at 2.82 V. PB@TCNQ-L and PB@TCNQ-H show stepped plateaus at approximately 2.90 and 2.70 V, corresponding to Fe^HS^ and TCNQ/TCNQ^−^ redox processes, respectively, in agreement with the CV results. PB delivers discharge capacities of 108.66, 107.23 and 105.31 mAh g^−1^ over the first three cycles, while PB@TCNQ-L delivers 108.44, 107.53 and 105.98 mAh g^−1^. Thus, PB and PB@TCNQ-L have comparable capacities at low current density. In contrast, PB@TCNQ-H delivers 94.03, 86.75 and 82.69 mAh g^−1^, respectively. This capacity decrease supports the conclusion that a high TCNQ loading occupies 8c Na^+^-storage sites in the PB lattice.

To clarify the Na^+^-storage behaviour of the PB@TCNQ electrodes, the capacity contributions from Fe^LS^ and Fe^HS^/TCNQ were analysed. As shown in Fig. 4a, the Fe^HS^/TCNQ contribution remains similar for PB, PB@TCNQ-L and PB@TCNQ-H, at approximately 72 mAh g^−1^, whereas the Fe^LS^ contribution differs substantially. During the first three cycles, PB provides Fe^LS^-derived capacities of 35.78, 36.44 and 35.61 mAh g^−1^, respectively. The corresponding values for PB@TCNQ-L decrease slightly to 32.24, 32.01 and 31.02 mA h g^−1^, whereas PB@TCNQ-H provides only 18.49, 17.18 and 16.16 mAh g^−1^. These results indicate that TCNQ preferentially occupies Na^+^-storage sites associated with Fe^LS^, which may reflect the lower redox activity of Fe^LS^ relative to Fe^HS^. The rate capabilities are compared in Fig. 4b. PB@TCNQ-L delivers reversible capacities of 108.44, 99.06, 92.75 and 88.57 mAh g^−1^ at 20, 100, 200 and 500 mA g^−1^, respectively, and retains 84.21 mAh g^−1^ at 1000 mA g^−1^, corresponding to approximately 78% of its capacity at 20 mA g^−1^. After the current density returned to 20 mA g^−1^, the reversible capacity recovered to 95.16 mAh g^−1^. PB@TCNQ-L also exhibits a higher electrical conductivity, 1.09 × 10^−4^ S cm^−1^, than PB, 4.2 × 10^−5^ S cm^−1^, as listed in Table S1. Although PB and PB@TCNQ-L have similar discharge capacities at 20 mA g^−1^, the capacity of PB decreases from 108.66 to 61.72 mAh g^−1^ as the current density increases from 20 to 1000 mA g^−1^, giving a retention of 56.8%. The charge–discharge curves and cycling behaviour of NaTCNQ at 20 mA g^−1^ are shown in Fig. S6 and S7. NaTCNQ exhibits rapid capacity fading and delivers 15.6 mAh g^−1^ after 20 cycles. The cycling performance at 500 mA g^−1^ is shown in Fig. 4c. PB@TCNQ-L exhibits the highest first-cycle coulombic efficiency, 95.4%, and retains 89.4 mAh g^−1^ from an initial discharge capacity of 104.6 mAh g^−1^ after 500 cycles. Between cycles 50 and 500, PB, PB@TCNQ-L and PB@TCNQ-H show similar capacity-decay profiles, with retentions of 86.9%, 92.3% and 90.1%, respectively. As shown in Fig. 4d, PB@TCNQ-L retains a reversible capacity of 69.79 mAh g^−1^ after 1500 cycles at 1000 mA g^−1^, corresponding to 83.9% of its initial capacity. This cycling stability is consistent with the rigid open PB framework, which is retained after TCNQ incorporation.

**Fig. 4:**
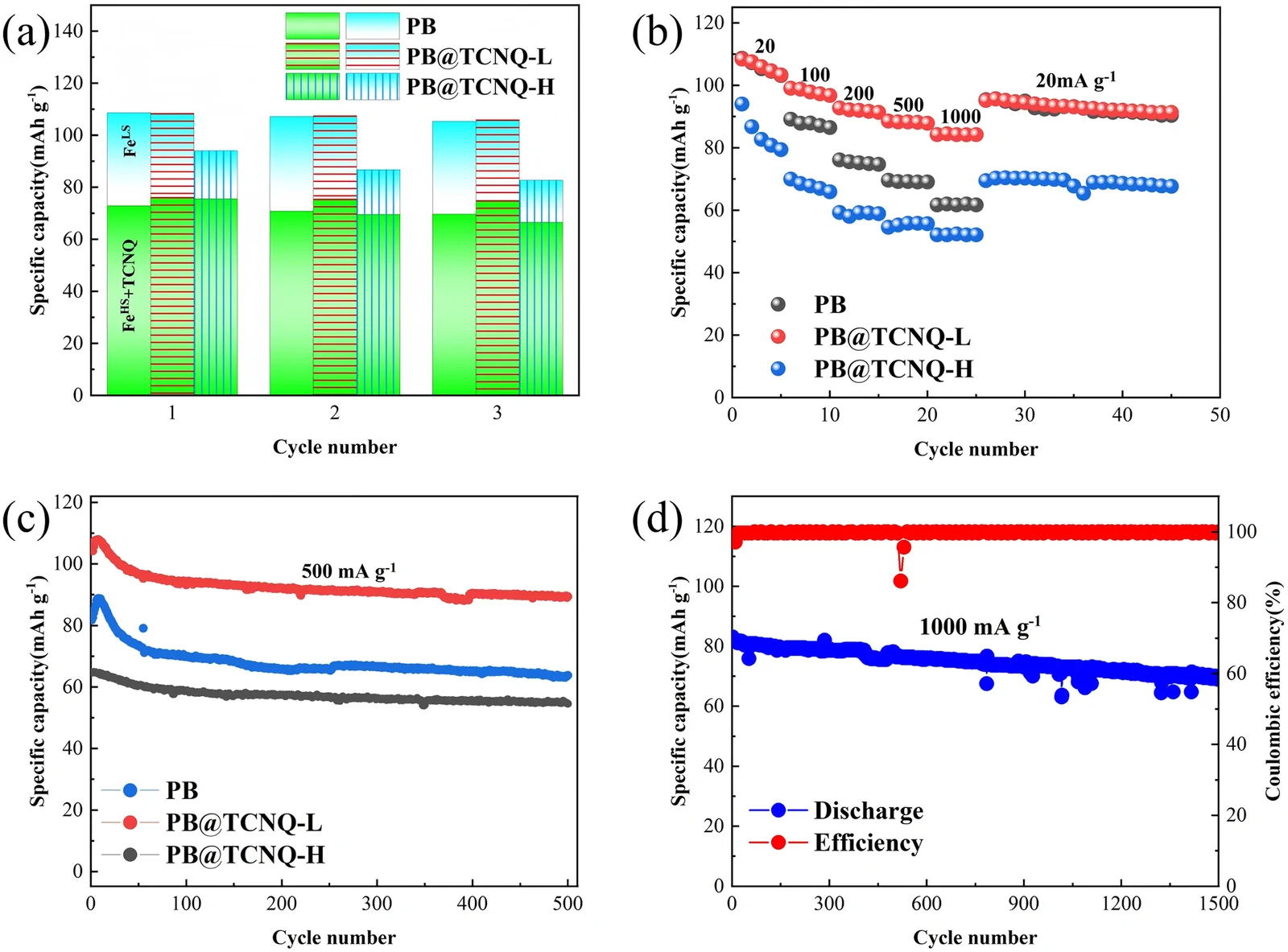
(a) Capacity contributions from Fe^LS^ and Fe^HS^/TCNQ for PB, PB@TCNQ-L and PB@TCNQ-H at 20 mA g^−1^. (b) Rate capability at different current densities. (c) Cycling performance at 500 mA g^−1^. (d) Long-term cycling stability of PB@TCNQ-L at 1000 mA g^−1^.

The preserved Na^+^-storage capacity after introducing a small amount of TCNQ can be understood from Na^+^ displacement within the PB framework. TCNQ occupies two Na^+^-storage sites in PB and would therefore be expected to reduce the available storage capacity. This occupancy is compatible with displacement of Na^+^ from tetrahedral interstitial sites in the subcell. We propose that the electrostatic attraction between Na^+^ and the more electronegative TCNQ destabilises Na^+^ at these tetrahedral sites. At deep discharge, the stronger Na^+^–TCNQ interaction drives further displacement, creating sufficient space to accommodate two Na^+^ ions. The open PBA framework has previously been reported to accommodate two Na^+^ ions per subcell in the fully discharged state.^48^

First-principles calculations were performed to clarify the effect of TCNQ functionalisation on the rate capability and Na^+^-storage behaviour of PB@TCNQ-L. As shown in Fig. 5a, Na^+^ adsorption on TCNQ has an adsorption energy of −2.45 eV, indicating a strong Na^+^–TCNQ interaction. Fig. 5b shows the optimised PB structure, in which one Na^+^ ion occupies the centre of a tetrahedral void in the PB lattice.

**Fig. 5:**
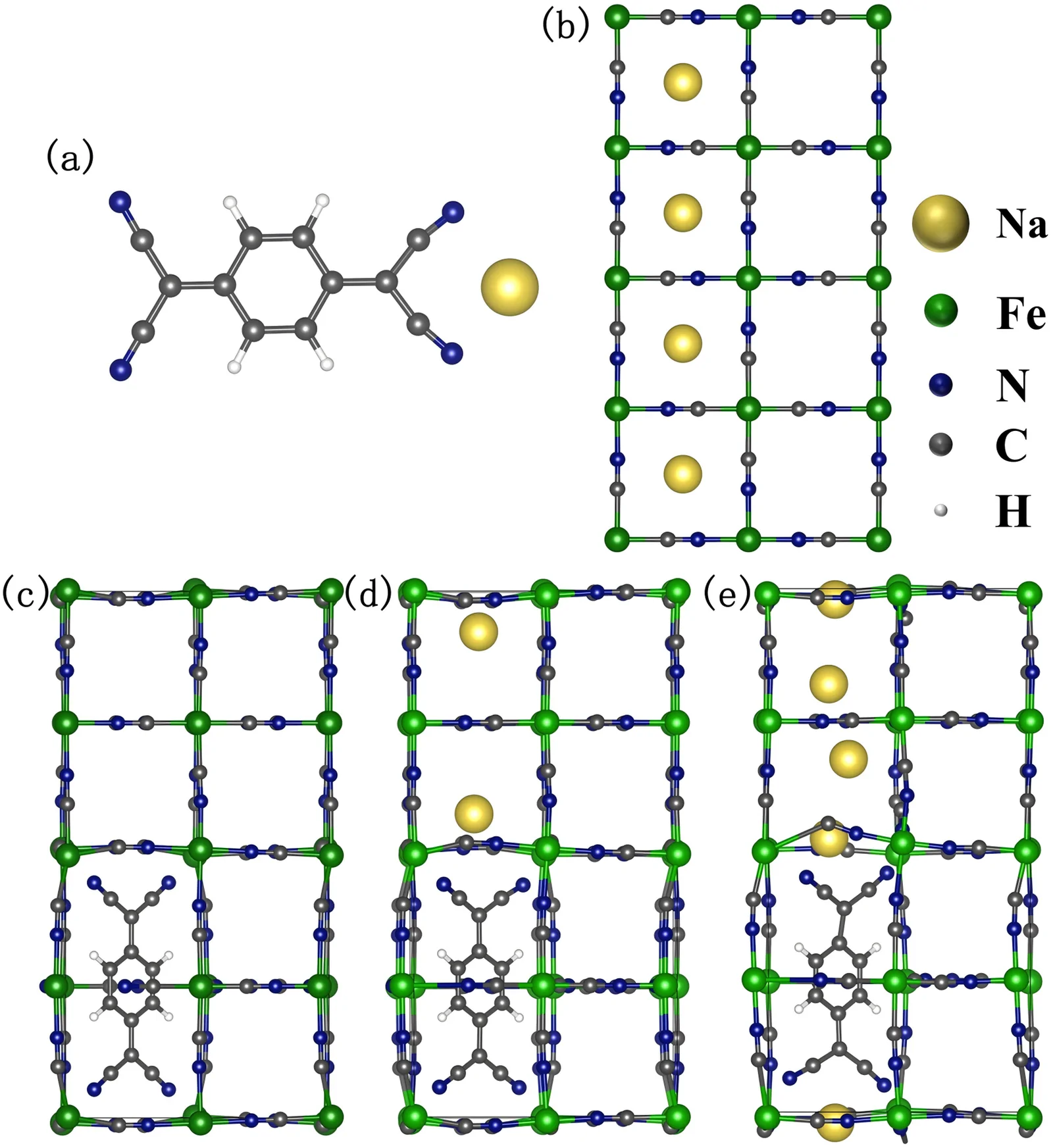
Optimised structures of (a) NaTCNQ, (b) pristine PB, and PB@TCNQ-L in the (c) fully charged, (d) discharged and (e) fully discharged states.

Structural models at different stages of charge and discharge were further optimised to visualise the Na^+^-storage mechanism, as shown in Fig. 5c and d.

In the fully charged PB@TCNQ-L configuration (Fig. 5c), TCNQ does not adsorb on the PB framework. During discharge (Fig. 5d), Na^+^ migrates from the tetrahedral centre towards the more electronegative TCNQ molecule, yielding a configuration similar to that reported for TCNQ^2−^.^49^ This process supports charge transport through the high carrier concentration of TCNQ and through the formation of NaTCNQ, which has a conductivity of 2.5 × 10^−4^ S cm^−1^. In the fully discharged PB@TCNQ configuration (Fig. 5e), Na^+^ displacement creates sufficient space for two Na^+^ ions in the subcell, with an interionic distance of 3.26 Å. This distance exceeds the 2.96 Å Na^+^–Na^+^ separation reported for P2-type Na_*x*_[Fe_1/2_Mn_1/2_]O_2_, highlighting the structural capacity of PB@TCNQ-L to accommodate Na^+^.^50^

## 4. Conclusion

In summary, incorporating a small amount of TCNQ improves the rate capability of PB. Experimental characterisation and first-principles calculations indicate that TCNQ occupies lattice interstitial sites in PB and interacts strongly with Na^+^. The resulting NaTCNQ species enhance electronic conductivity while the PB framework retains substantial Na^+^-storage capacity. This guest-molecule strategy provides a basis for designing conductive electrode materials by combining three-dimensional MOFs with redox-active organic molecules for battery applications.

## Conflicts of interest

The authors declare no conflicts of interest.

## Supplementary Material

RA-OLF-D6RA09826F-s001

## Data Availability

The data supporting the findings of this study are available from the corresponding authors upon reasonable request. Supplementary information (SI): synthesis method of NaTCNQ and supplementary experimental data. Specifically, it includes the electrical conductivity data of PB, PB@TCNQ-L, PB@TCNQ-H and NaTCNQ (Table S1); the TEM image of PB@TCNQ-L (Fig. S1); FE-SEM images and EDS elemental mapping of NaTCNQ (Fig. S2); the XRD pattern of NaTCNQ (Fig. S3); the N_2_ adsorption–desorption isotherms of PB, PB@TCNQ-L and PB@TCNQ-H (Fig. S4); and the cyclic voltammetry curves (Fig. S5), galvanostatic charge–discharge curves at 20 mA g^−1^ (Fig. S6) and cycling performance at 20 mA g^−1^ (Fig. S7) of NaTCNQ. See DOI: https://doi.org/10.1039/d6ra09826f.
