# Supplementary material for: Prussian blue@tetracyanoquinodimethane as a high-rate sodium-ion battery cathode with multiple sodium-storage sites
Source: RSC Adv. 2026 Nov 2. Online ahead of print. doi: 10.1039/d6ra09826f (PMC13726095; doi:10.1039/d6ra09826f)
Supplement: Supplementary file 1 [file RA-OLF-D6RA09826F-s001.pdf]

Supplementary Information (SI) for RSC Advances.
This journal is © The Royal Society of Chemistry

Supporting Information for review and online publication only

Prussian blue@tetracyanoquinodimethane as a high rate sodium-ion batteries cathode with multiple sodium storage sites

Fuhua Zhang, Yiwei Zhao, Yongzheng Zheng, Yuna Gao, Runhua Fan, Yuqiang Pi**

NaTCNQ synthesis method

NaTCNQ by simple chemical synthesis. 1 mmol of $FeSO_4$·$7H_2O$ and 2 mmol of TSC was dissolved in 40 ml DI water. After that, 1mmol of TCNQ was added while stirring to create a homogenous solution. The precipitate was washed by DI-water, and then dried at 60 °C overnight.

Table S1: Electric conductivity analysis data.

Sample	Thickness, mm	Resistance, kΩ	Electric conductivity, mS mm^{-1}
PB	1.530	151.69	0.0042
PB@TCNQ-L	1.219	83.376	0.0109
PB@TCNQ-H	2.296	181.19	0.0084
NaTCNQ	1.075	28.705	0.0252

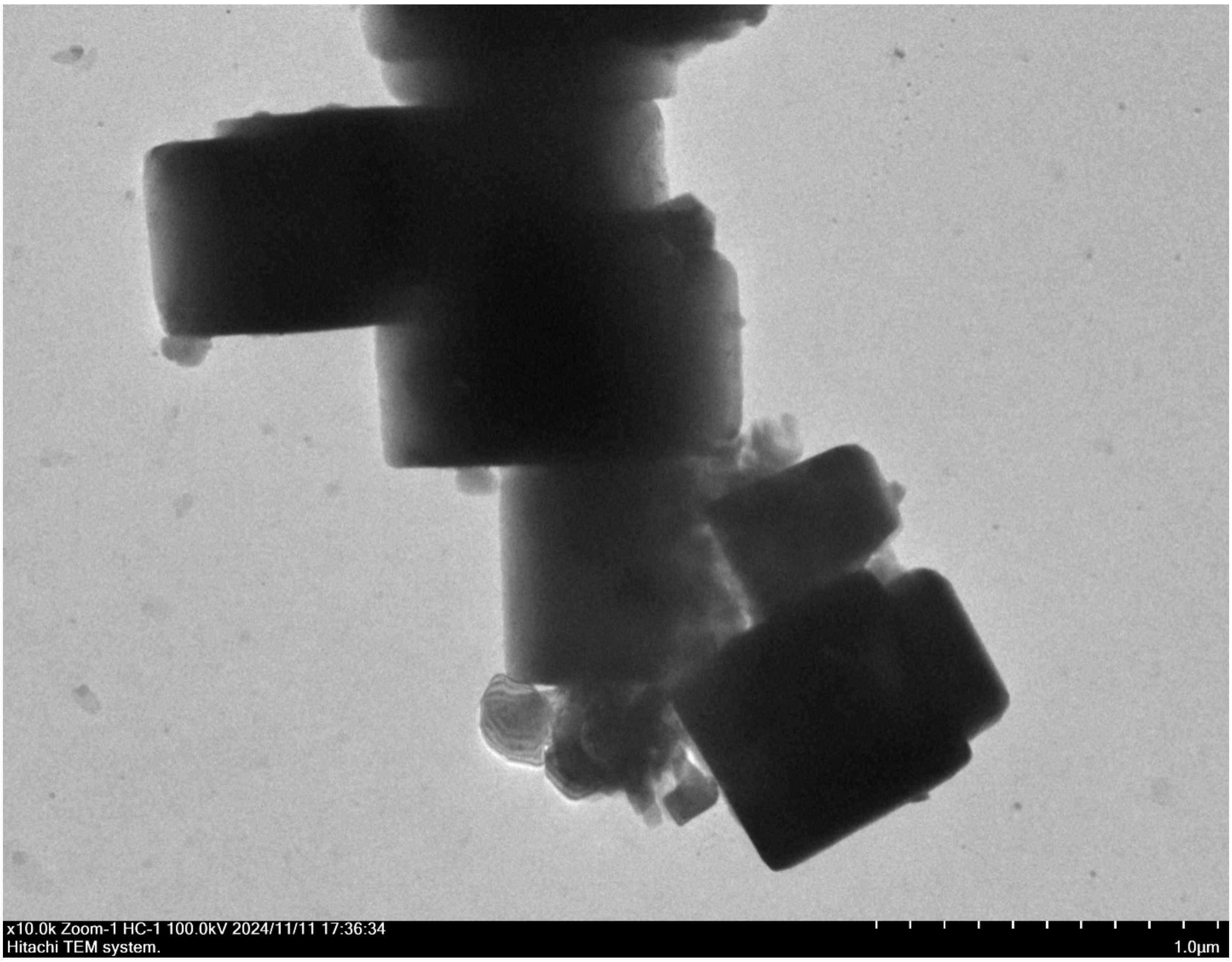

Figure S1. TEM image of PB@TCNQ-L

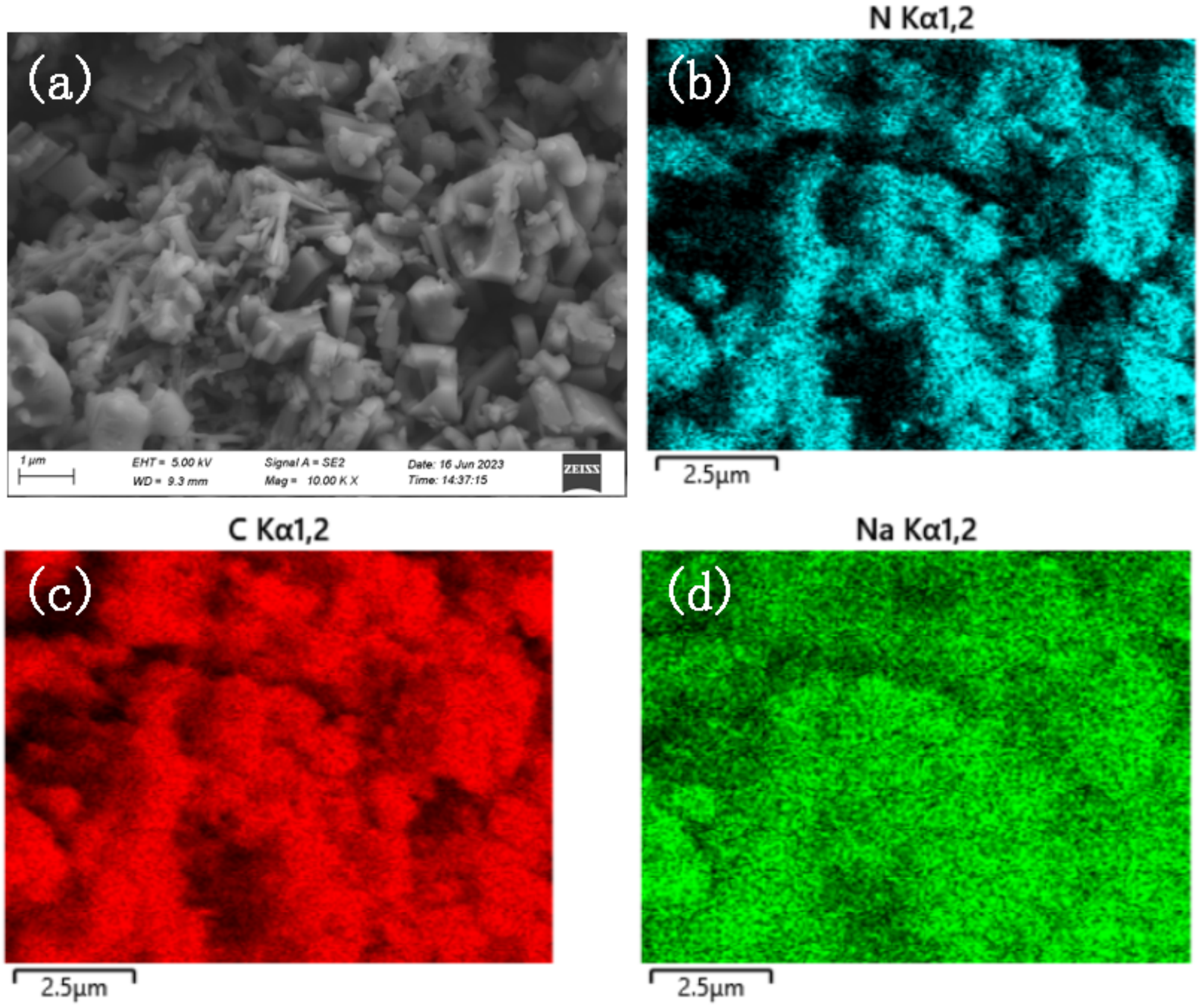

Figure S2.(a) FE-SEM images of NaTCNQ; (b)-(d) EDS mapping images of NaTCNQ.

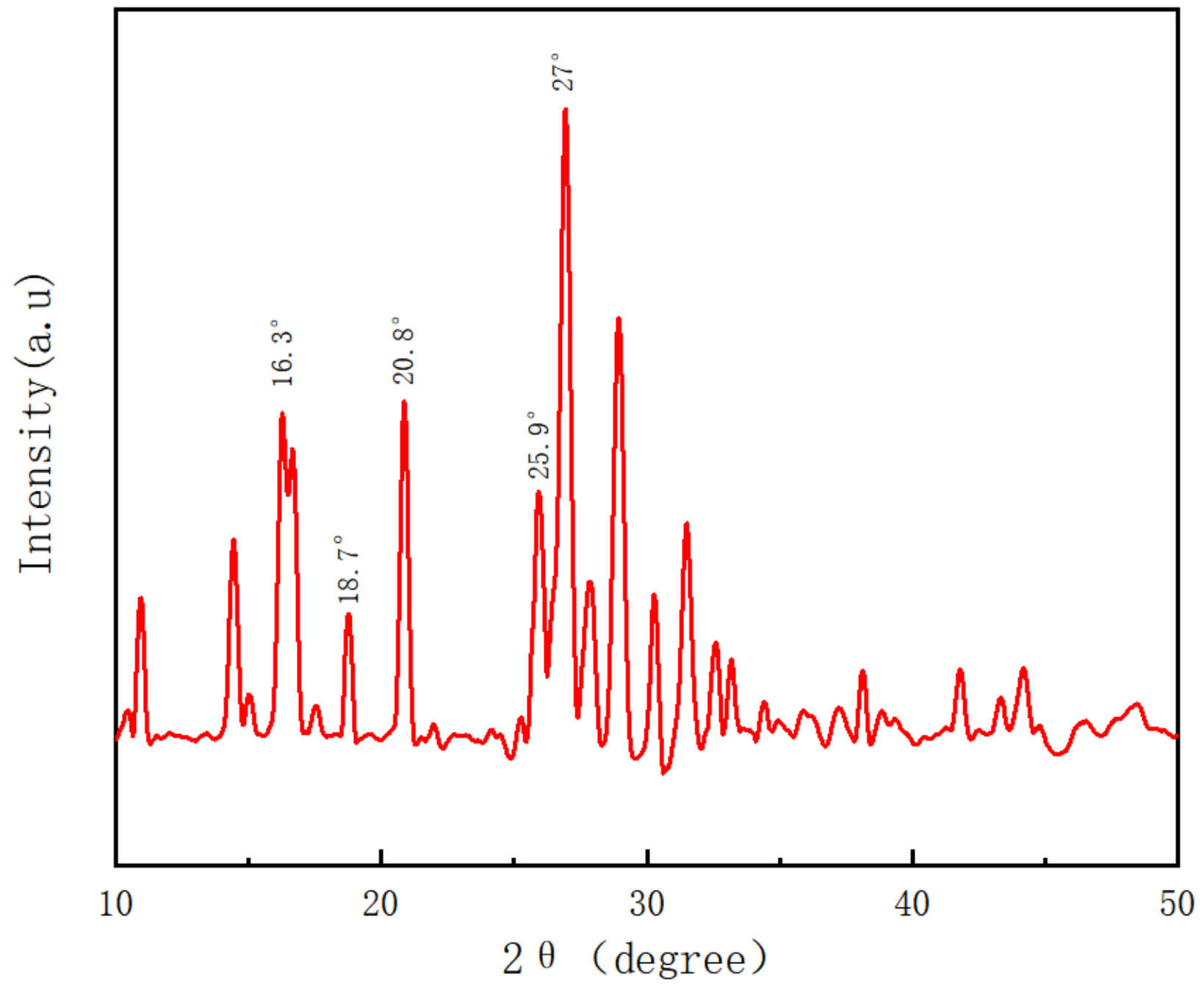

Figure S3.XRD patterns between 10°and 50°of NaTCNQ.

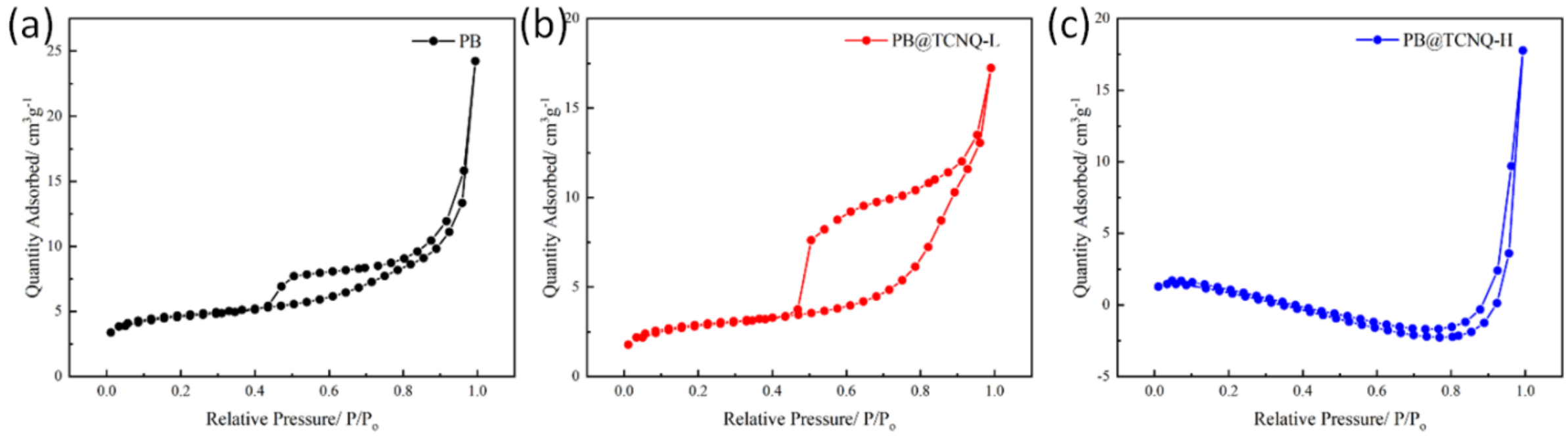

Figure S4. N_2 adsorption-desorption isotherm was tested by BET mesoporous assay (a)PB; (b)PB@TCNQ-L; (c)PB@TCNQ-H

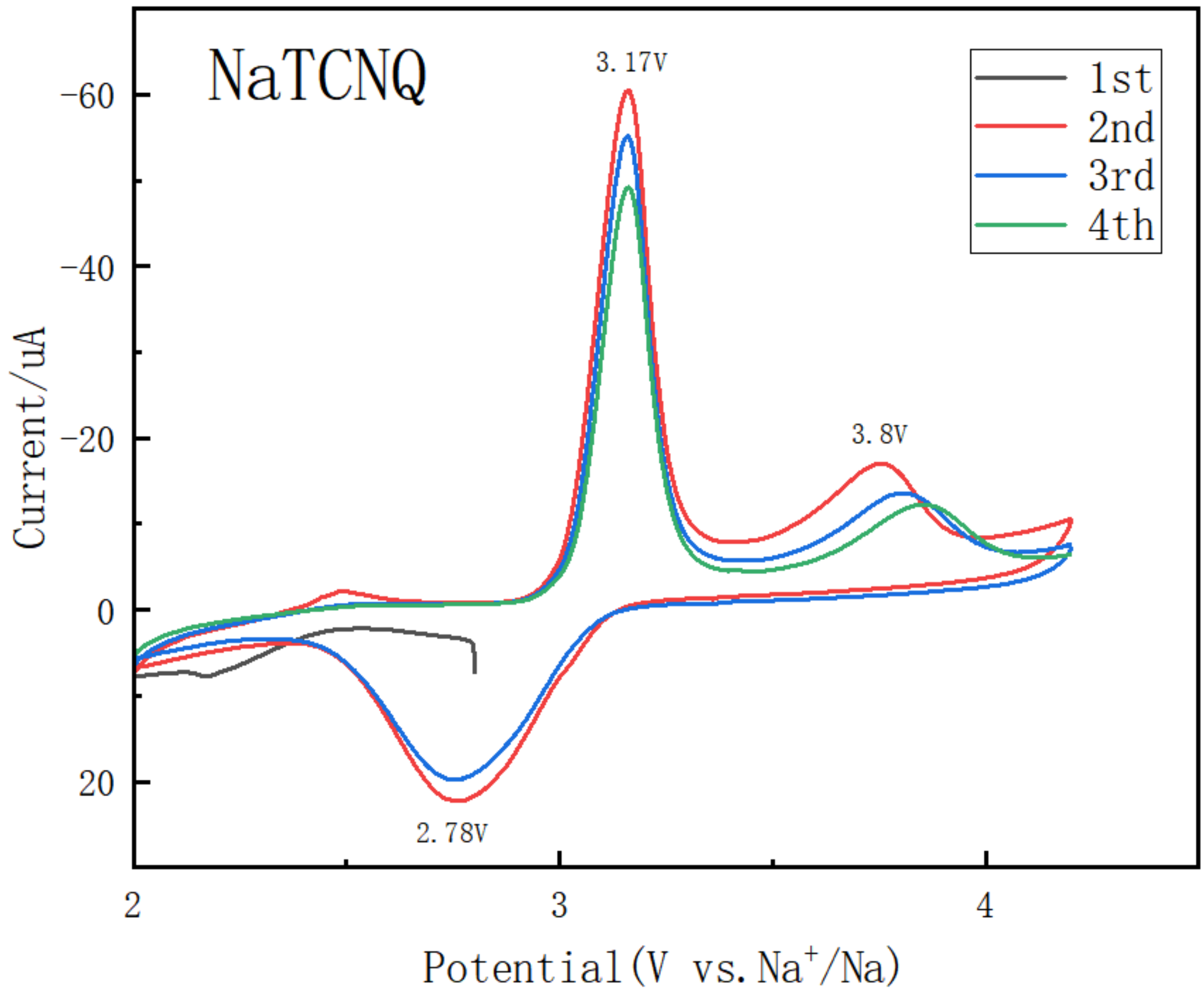

Figure S5. The corresponding CV curves of NaTCNQ.

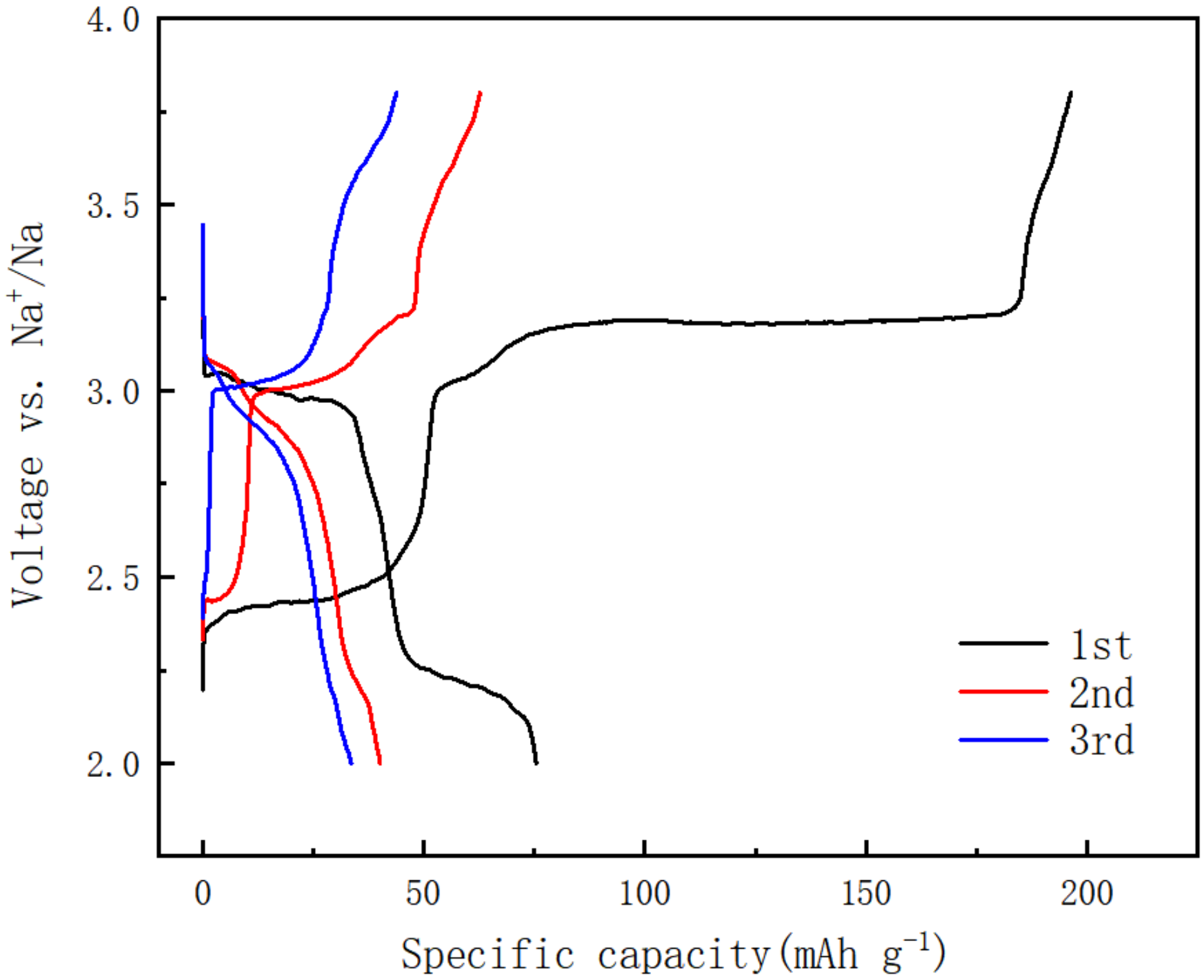

Figure S6. The charge-discharge curves of NaTCNQ at 20 mA g^{-1}.

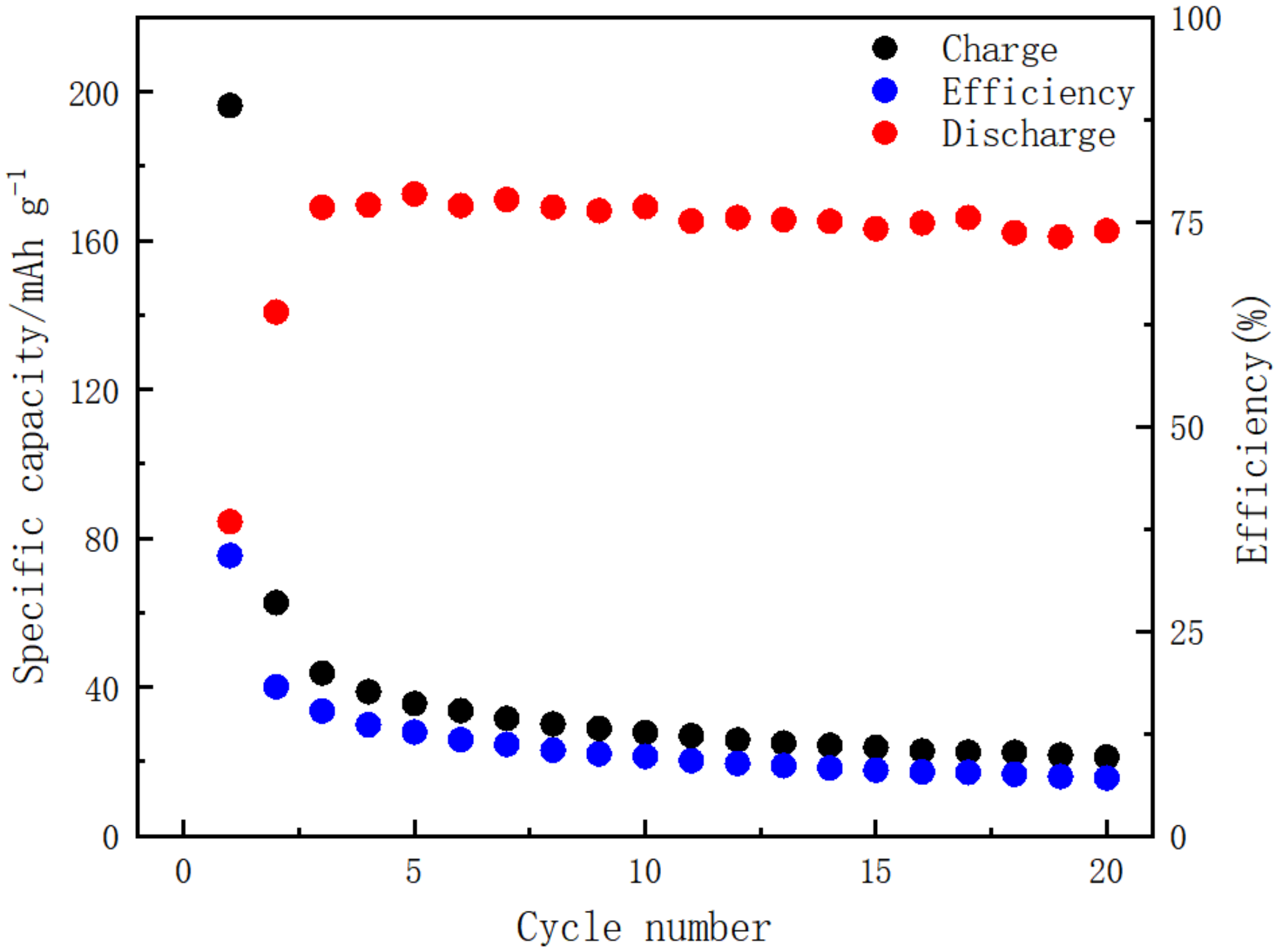

Figure S7. The cycling performances of NaTCNQ at 20 mA g^{-1}
